# Methylosystem for Cancer Sieging Strategy

**DOI:** 10.3390/cancers13205088

**Published:** 2021-10-12

**Authors:** Shotaro Tatekawa, Ken Ofusa, Ryota Chijimatsu, Andrea Vecchione, Keisuke Tamari, Kazuhiko Ogawa, Hideshi Ishii

**Affiliations:** 1 Department of Radiation Oncology, Osaka University Graduate School of Medicine, Suita, Yamadaoka 2-2, Osaka 565-0871, Japan; s_tatekawa@radonc.med.osaka-u.ac.jp (S.T.); tamari@radonc.med.osaka-u.ac.jp (K.T.); 2 Department of Medical Data Science, Center of Medical Innovation and Translational Research, Osaka University Graduate School of Medicine, Suita, Yamadaoka 2-2, Osaka 565-0871, Japan; oof21443@ideacon.co.jp (K.O.); rchijimatsu@cfs.med.osaka-u.ac.jp (R.C.); 3 Food and Life-Science Laboratory, Prophoenix Division, Idea Consultants, Inc., Osaka 559-8519, Japan; 4 Department of Clinical and Molecular Medicine, University of Rome “Sapienza”, Santo Andrea Hospital, Via di Grottarossa, 1035-00189 Rome, Italy; andrea.vecchione@uniroma1.it

**Keywords:** methylation, one-carbon metabolism, RNA, nicotinamide, cancer-associated fibroblasts

## Abstract

**Simple Summary:**

Cancer metabolism plays a fundamental role in cancer biology in the tumor microenvironment. Under the reaction of one-carbon metabolism-dependent S-adenosylmethionine, methylation defines a biologically malignant phenotype of cancer. As methylation is closely associated with malignant phenotypes in various cell types, including epithelial cancer cells, immune cells, and cancer-associated fibroblasts, in tumor tissues, a cancer sieging strategy targeting the “methylosystem” may be an effective therapeutic approach against refractory cancers.

**Abstract:**

As cancer is a genetic disease, methylation defines a biologically malignant phenotype of cancer in the association of one-carbon metabolism-dependent S-adenosylmethionine (SAM) as a methyl donor in each cell. Methylated substances are involved in intracellular metabolism, but via intercellular communication, some of these can also be secreted to affect other substances. Although metabolic analysis at the single-cell level remains challenging, studying the “methylosystem” (i.e., the intercellular and intracellular communications of upstream regulatory factors and/or downstream effectors that affect the epigenetic mechanism involving the transfer of a methyl group from SAM onto the specific positions of nucleotides or other metabolites in the tumor microenvironment) and tracking these metabolic products are important research tasks for understanding spatial heterogeneity. Here, we discuss and highlight the involvement of RNA and nicotinamide, recently emerged targets, in SAM-producing one-carbon metabolism in cancer cells, cancer-associated fibroblasts, and immune cells. Their significance and implications will contribute to the discovery of efficient methods for the diagnosis of and therapeutic approaches to human cancer.

## 1. Introduction

**Figure 1:**
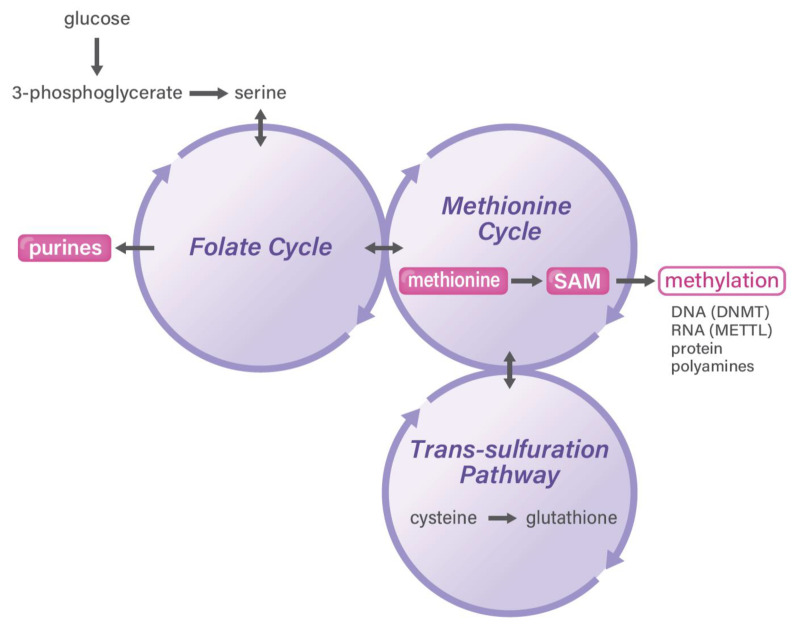
One-carbon metabolism in cancer. One-carbon metabolism is a mechanism that is coupled with three reactions of the folate cycle, methionine cycle, and trans-sulfuration pathway. The circled and highlighted areas indicate those playing a role in cancer and stem cells, as highlighted in the text. DNMT, DNA methyltransferase; METTL, adenosine-methyltransferase.

Cancer consists of highly heterogeneous tissues and comprises mutation-driven biologically malignant cancer cells, immune cells, cancer-associated fibroblasts (CAFs), and blood vessel endothelial cells [1]. The tumor microenvironment (TME) contains components that adapt to the originally occurring primary tumor sites because of their anatomical location, immunosuppressive environment, and unique metabolism. Moreover, TME refers to metastatic sites in distant organs [2]. Although the tumor mutation burden is defined by the total number of somatic mutations in cancer cell DNA, research has elucidated that metabolism plays a fundamental role in cancer biology in TME [3]. Research has demonstrated that interactions among cancer cells, immune cells, and CAFs emerge via metabolites based on biomass and the energy production of one-carbon metabolism (Figure 1) [4]. This mechanism is an attractive target for efficient cancer drug discovery. Further, as an aspect of cancer prevention during the early stages of cancer, an association between food and nutrient intake has emerged. A recent study from a broad review of meta-analyses of observational studies evaluating the strength and validity of the evidence for factors associated with the risk of developing or dying from 11 primary cancers indicated that the consumption of dairy products, milk, calcium, and whole grains is inversely associated with cancer risk, suggesting an effective outcome of nutrient intake [5]. Given that folates are important nutrients obtained through the diet, recent studies indicated that the dietary intake of one-carbon metabolism-related nutrients is associated, inversely or positively, with pancreatic cancer [6] and melanoma risk [7], suggesting that metabolites are involved in intra- or intercellular metabolism under the influence of essential nutrients. This finding might be beneficial for cancer medicine and aging research, although it warrants further investigation [8].

## 2. Genome and Metabolome

Recent developments in next-generation sequencing have enabled studies at the single-cell level. Spatial transcriptome analysis, a new method for obtaining positional information on cells in cancer via single-cell analysis, was applied to various clinical specimens to investigate the transcriptome at the intracellular and intercellular levels [9]; however, measuring metabolites in single cells remains challenging [10]. As a metabolite of one-carbon metabolism, SAM plays a critical role in the methylation of various targets, including DNA, RNA, and proteins, and of several other metabolites [11]. Methylated substances are involved in intracellular metabolism in TME, but some can also be secreted to affect other substances via intercellular communication [12]. In this review, we focused on the elucidation of intercellular and intracellular communications between upstream regulatory factors and/or downstream effectors that affect the epigenetic mechanism involving the transfer of a methyl group from SAM onto the specific positions of nucleotides or other metabolites in TME as a whole tissue; in TME, various cells, including epithelial cancer cells, CAFs, endothelial cells, immune cells, and others, are involved and track important products. To understand the spatial heterogeneity, we refer to this as the “methylosystem.” The significance and implication of this review will contribute to the discovery of efficient methods for diagnosing and devising therapeutic approaches to human cancer. Understanding the cellular and molecular mechanisms underlying the methylosystem could be a novel strategy for disrupting cancer cell interactions and contribute to the development of efficient and safe therapeutic strategies to treat cancer. Furthermore, research findings can be used as cancer diagnostic tools for developing precision medicine by precisely predicting and monitoring cancer therapy outcomes.

## 3. Oncogenes and TME-Driven Metabolism Alterations

Otto Heinrich Warburg was awarded the 1931 Nobel Prize in physiology for his “discovery of the nature and mode of action of the respiratory enzyme” [13]. Warburg was rewarded as his discovery, termed the Warburg effect, explained that cancer cells largely depend on aerobic glycolysis, whereas, in sharp contrast, normal differentiated cells primarily rely on oxidative phosphorylation in mitochondria to generate energy and biomaterials needed for cellular processes [14].

Recent studies have indicated that cancers indeed exhibit the Warburg effect, an increased uptake and conversion of glucose to lactate, but cancer cells are also associated with alterations in glutamine and fatty acid metabolism [15]. In this malignant mechanism, the *MYC* oncogene contributes to alterations in cellular metabolism to facilitate tumorigenesis by altering nucleotide metabolism and DNA replication induced by the attenuated expression of E2F1; hypoxia-inducible transcription factor 1; lactate dehydrogenase A; and several microRNAs, such as *miR-23a/b*, to increase the protein expression of glutaminase [15]. In addition to the regulation of gene expression at the transcriptional level, research has revealed the significance of the ubiquitin-proteasome system (UPS), which controls various signaling factors in the glycolysis pathway via ubiquitination or deubiquitination [16]. As a result of UPS, deubiquitination acts as either a tumor-promoting oncoprotein or as a tumor-restricting suppressor protein [16].

Cellular metabolism in cancer cells is altered in terms of the mechanism of oxidative phosphorylation within the mitochondria. First, in TME, hypoxia promotes the isocitrate dehydrogenase (IDH)-dependent carboxylation of α-ketoglutarate to citrate, which further contributes to the malignant phenotype of cancer cells; these phenotypes include rapid cell growth, extended survival under hypoxia, malnutrition, and therapeutic resistance [17]. Second, a study on malignant cells found that the reduced glutamine metabolism by IDH1 mediates lipogenesis under a hypoxic TME; this indicates a critical role for the oxidative process in regulating carbon use for producing acetyl coenzyme A, the central biosynthetic precursor that supports fatty acid synthesis and protein acetylation in mammalian cells [18]. Another study on colorectal cancer indicated that the metabolism of the IDH-dependent carboxylation of α-ketoglutarate to citrate was altered by the oncometabolite D-2-hydroxyglurate (HG), which directly induced the epithelial-mesenchymal transition and was associated with the distant metastasis of cancer cells [19]. A mathematical analysis of cancer patient data predicted that imbalanced IDH1/2 expression is associated with the 2-HG-inactivating β-oxygenation pathway in colorectal cancer [20]. Third, given that the *KRAS* oncogene is among the most frequently mutated genes in pancreatic cancer, a study on the metabolism of pancreatic cancer cells indicated that they rely on a distinctive pathway. Via this pathway, glutamine supports pancreatic cancer growth via a *KRAS*-regulated metabolic pathway; in this pathway, it can be converted into oxaloacetate by aspartate transaminase and this oxaloacetate is further converted into malate and then pyruvate, which contributes to an increase in the NADPH/NADP+ ratio and leads to the maintenance of the cellular redox state [21]. It has been suggested that the essentiality of this pathway in pancreatic ductal adenocarcinoma and the fact that it is critical in normal cells may provide novel therapeutic approaches for treating these refractory tumors [21]. By contrast, in a study on colorectal cancer, cases with *KRAS* mutations demonstrated a different role of this oncogene in the malignant phenotype, suggesting a role of KRAS in the metabolic adaptation mechanism to nutritional stress in colorectal cancer [22]. Further, in colorectal cancer, the V600E mutation in the *BRAF* oncogene was found to be involved in AMP-activated protein kinase-mediated autophagy and therapeutic resistance in cancer cells [23].

## 4. Methionine and One-Carbon Metabolism Pathway in Cancer

As somatic stem cells, hematopoietic stem cells play a functional role at the center of hematopoiesis, and specific metabolic changes in hematopoietic stem and progenitor cells (HSPCs) have been linked with the induction of alterations in myelopoiesis in the bone marrow as well as with HSPC dysfunction in aging and clonal hematopoiesis [8]. HSPC function is regulated by metabolic processes during various stimuli, such as immunologic and inflammatory responses [24]. Folate metabolism is among the most functionally important metabolic processes of hematopoiesis and the immune response, which is consistent with the fact that hematopoiesis and the immune response are the most proliferative processes in the body [25]. For example, long-term dietary folate deficiency can induce macrocytic anemia. Antifolate metabolism antagonists against hematopoietic malignancies and other solid cancers are the most important methods as chemotherapy to reduce cancer-specific metabolism and inhibit the proliferation of cancer cells [26]. Although recent research has shown that immune cells are sensitive to exposure to conventional antifolate therapies, which can limit the effective doses required to eradicate cancer cells, the antifolate reagent methotrexate is an anchoring drug in chronic arthritis and systemic lupus erythematosus [27]. It has emerged that more sensitive antifolate metabolism antagonists in immune cells are necessary for clinical use.

Diet is a major source of one-carbon units and includes three groups: (1) glucose and its glycolysis product, serine; (2) methionine cycle products (such as methionine and choline); and (3) glycine, which can be derived from threonine via the reaction of L-threonine dehydrogenase (TDH) in rodents, but not in humans, as the human *TDH* encodes a pseudogene without functional catalytic activity [25]. Moreover, histidine can be incorporated into one-carbon units in an alternative pathway.

**Figure 2:**
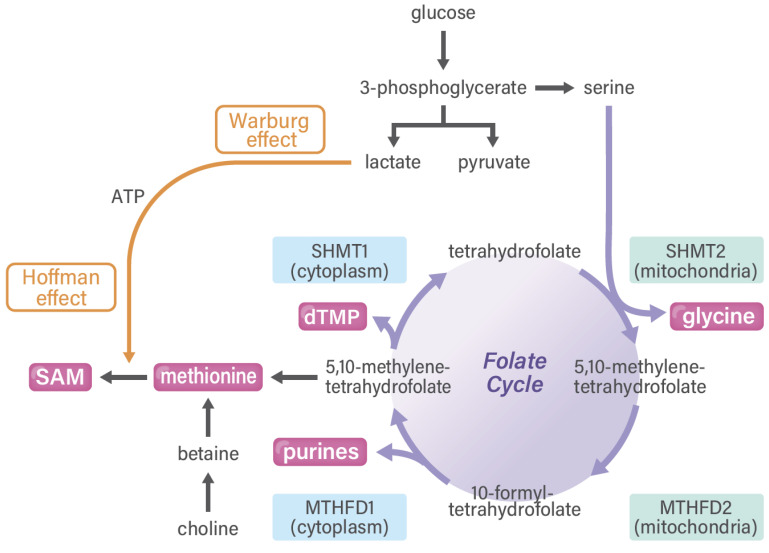
Folate cycle and SAM in cancer. The Warburg and Hoffman effects collaborate with each other via one-carbon metabolism. MTHFD and SHMT are druggable targets for cancer treatment. MTHFD, methylenetetrahydrofolate dehydrogenase; SAM, S-adenosylmethionine; SHMT, serine hydroxymethyltransferase.

The one-carbon pathway differs among various cell types (Figure 2). First, in cancer cells, which proliferate quickly, one-carbon metabolism is activated and plays a role in the production of purine, which is a precursor of nucleotides (such as DNA and RNA). In cancer cells, glucose can be predominantly used and gives rise to serine and purines. In a study on clinical samples, the enzyme status of one-carbon folate metabolism was shown to predict the survival rate of patients with gastrointestinal cancer; this finding provides a rationale for this pathway as certain anticancer drug targets [28], such as methylenetetrahydrofolate dehydrogenase (MTHFD) 2 [29], suggesting the druggability of one-carbon metabolism in cancer diagnostic and therapeutic approaches [30]. Previous reports have indicated that >80% of cancer cells eventually depend on the uptake of extracellular methionine and that cancer cells can rapidly synthesize methionine from homocysteine, which is consistent with the general requirements of cancer cells for methionine for altered metabolic flux via a pathway linked to SAM usage [31]. The high demand or “addiction” of cancer cells to exogenously provided methionine is not caused by the cancer cells’ inability to synthesize methionine but rather by their high demands of methionine-derived metabolites [32], including processes involving Cdc6 and prereplication complexes [33], nucleoside metabolism and polyamine synthesis [34], and cell cycle arrest in G1 involving p38 mitogen-activated protein kinase [35]. The dependence of cancer cells on methionine is referred to as the methionine stress sensitivity of cancer cells or the Hoffman effect [31]. Of note, Sugimura et al. provided the *in vivo* evidence of tumor dependency on dietary methionine, demonstrating that tumor growth in rats is significantly affected by the restriction of individual amino acids such as methionine [36]. However, the magnitude of the dependence of SAM synthesis on folate metabolism or betaine on cancer cells remains unclear.

**Figure 3:**
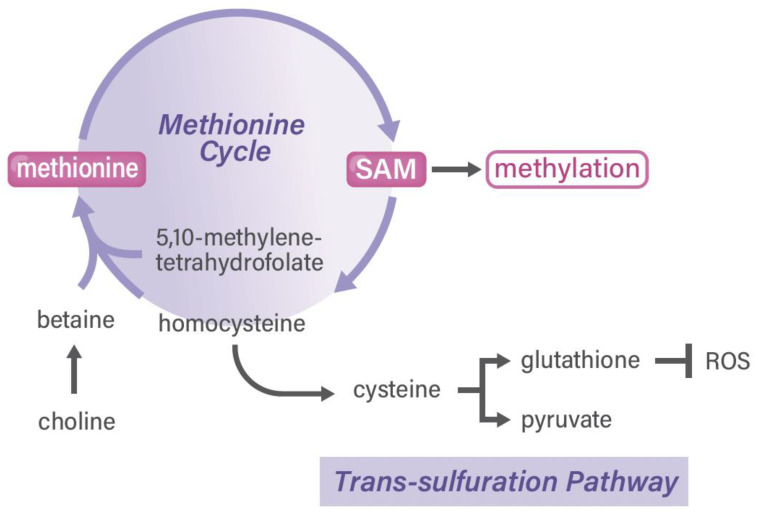
Methionine cycle and SAM in cancer. ROS, reactive oxygen species; SAM, S-adenosylmethionine.

Second, glucose-dependent serine synthesis and folate metabolism are vital for the production of purine in somatic stem cells, which is required for cancer and embryonic development [25]. A previous study on an animal model of *MTHFD2−/−* embryos indicated that MTHFD2 has functional importance in hematopoietic lineages, including immune cells [37]. In TME, T cells are required for rapid proliferation and prompt transcriptional responses to various stimuli, which necessitate rapid one-carbon metabolism. In addition, a biochemical study on T cell function [38] and a clinical investigation on gastrointestinal cancer [28] noted large changes in the gene expression of genes in the serine hydroxymethyltransferase (SHMT)2 to MTHFD2 metabolic pathway. A study on immune cells with the antioxidant N-acethylcycteine indicated that the activation of T cells requires both the generation of a one-carbon unit and redox defense (Figure 3) [39], suggesting that T cell activation is linked to the trans-sulfuration pathway in one-carbon metabolism [4] and that there is a great need for developing MTHFD2-specific reagents [25]. Nonetheless, one-carbon metabolism and drug discovery in cancer stem cells (i.e., a fraction of stemness-possessing cancer cells in whole-tumor tissues) remains to be completely understood. Of note, the authors of a rat study reported the indispensable role of several amino acids in maintaining somatic stem cells, which is indicative of the finding that depleting dietary valine permits nonmyeloablative mouse hematopoietic stem cell transplantation [40]. In previous studies, some dietary factors, including casein [41], folate, and amino acids [42], were found to play important roles in the recovery after granulocytopenia in rats. Research has further extended the use of compounds in long-term ex vivo hematopoietic stem cell expansion, which will enable nonconditioned transplantation [43].

Third, SAM can be synthesized not only from folate in differentiated somatic cells but also from betaine, which is derived from choline. As mentioned above, in sharp contrast to cancer cells, noncancerous somatic cells can use betaine to produce sufficient SAM to maintain the homeostasis of the methylation of nucleotides and protein in cells [25]. Cancer cells’ requirement for methionine is purported to be caused by the high demand of SAM and one-carbon metabolism-related metabolites in cancer cells. It has been reported that SAM is involved in ornithine decarboxylase in the polyamine pathway of cancer stem cells in osteosarcoma [44] and cervical cancer [45], which is useful for drug discovery in targeting esophageal cancer [46]. Furthermore, polyamine flux suppresses histone lysine demethylases and enhances *ID1* expression in cancer stem cells [47]. Taken together, the one-carbon metabolism pathway plays a role in different cell types, although understanding the dietary factors warrants further studies.

## 5. RNA Methylation Pathways in Cancer

**Figure 4:**
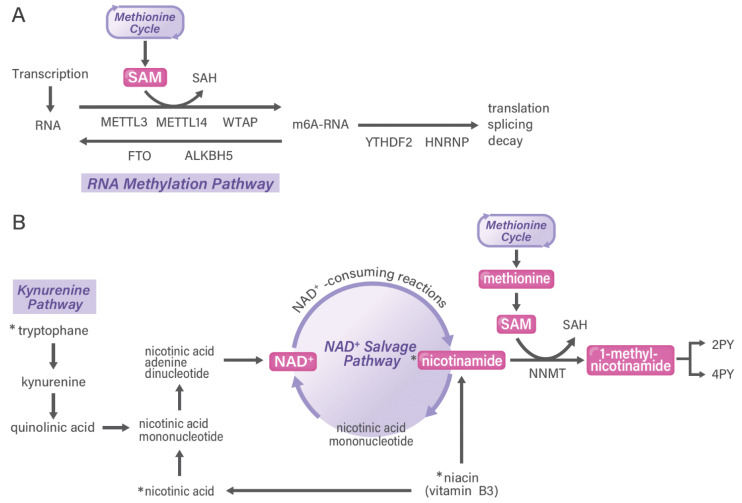
RNA methylation pathway and NAD+ salvage pathway in cancer. In tumor tissues, heterogeneous cell populations exert differential functions of the RNA methylation pathway (**A**) and the NAD+ salvage pathway (**B**), which are examples of the methylosystem. Nicotinamide metabolism is shown as the NAD+ salvage pathway in the correlation of the kynurenine pathway and the one-carbon pathway. Although NAD+ can be synthesized from tryptophan in the kynurenine pathway, this metabolic pathway is less effective in humans than in mice. 2PY, N-methyl-2-pyridone-5-carboxamide; 4PY, N-methyl-4-pyridone-3-carboxamide; NAD+, nicotinamide adenine dinucleotide; SAM, S-adenosylmethionine; SAH, S-adenosyl-homocysteine. Asterisks indicate dietary intake. Asterisks (*) indicate ones from diet.

One-carbon metabolism is known to be associated with nucleotide methylation pathways, including for both DNA and RNA. Because DNA methylation is an important characteristic of cancer, it has been studied extensively and has broadened the scope of epigenetics as a functional assay to elucidate disease pathophysiology [48]. DNA methylation in liquid biopsies in blood has emerged in the initial diagnosis of cancer but also in the early detection of relapse after therapy [49]. In contrast to DNA modification being predominant in 5-methyl cytosine, RNA modifications occur in 6-methyl adenine (m6A) and in various positions of nucleotides, which characterize heterogeneous cancer types (Figure 4A) [50]. Measuring m6A in microRNAs, which are small noncoding RNAs, is useful for detecting the early stages and relapse phases of cancer [51].

Human N6-adenosine-methyltransferase complex catalytic subunit (METTL3) and METTL14 were shown to form a stable heterodimer core complex that functions in catalyzing m6A RNA methylation [52]. WT1-associated protein (WTAP) interacts with this complex and affects methylation [52]. The core complex of METTL3-14 with WTAP plays a role in writing epitranscriptome (RNA modification) codes [53]. By contrast, demethylases that reverse this methylation, termed erasers, have been identified: fat mass and obesity-associated protein [54] and α-ketoglutarate-dependent dioxygenase homolog 5 [55]. These modifications can be recognized to execute function by readers: heterogeneous nuclear ribonucleoproteins and YT521-B homology (YTH) N6-methyladenosine RNA binding protein 1 (YTHDF1). A study on colorectal cancer found that the expression of the m6A reader *YTHDF1* is controlled by the oncogene *c-myc* [56]. Taken together, one-carbon metabolism and its metabolites are involved in dynamic m6A modifications, which can be recognized by different binding proteins to exert the biologically malignant phenotypes of cancer.

## 6. Nicotinamide Adenine Dinucleotide (NAD+) Salvage Pathway in Cancer

In 1951, Cantoni first partially purified nicotinamide N-methyltransferase (NNMT) from rat liver [57] and subsequently discovered the structure of the cofactor SAM, which is an active methyl donor [58]. The enzymatic activity of NNMT is important for preventing the nicotinamide (NAM)-mediated inhibition of NAD+-consuming enzymes (such as poly-adenosine-diphosphate, ribose polymerases, and sirtuins) [59]. NNMT activity alteration has been reported in oral, stomach, colon, rectum, liver, pancreas, breast, bladder, prostate, ovary, and lung tumors as well as in glioma, lymphoma, and insulinoma [60,61,62]. The clinical significance of the involvement of NNMT was examined in a systematic review and meta-analysis [63], which indicated the prognostic value of NNMT expression in patients with solid tumors. Table 1 highlights the subsequent original reports and other basic research reports.

The involvement of gene expression via epigenetic regulation has been reported via the histone H3 lysine 9 demethylation mechanism [64]. In addition, NNMT has been shown to be involved in the epithelial-mesenchymal transition under the condition of glucose deprivation [65]. A study on gastric cancer showed that NNMT promotes the epithelial-mesenchymal transition [66]. NNMT downregulation also inhibits migration and the epithelial-mesenchymal transition in esophageal squamous cell carcinoma [67]. Moreover, another study on esophageal squamous carcinoma showed that NNMT is involved in metabolic reprogramming and promotes the Warburg effect [68]. NNMT and 1-methylnicotinamide (MNAM) are reportedly involved in the mechanism of inhibition of the apoptosis signal-regulating kinase 1-p38 MAPK pathway, resulting in increased colorectal cancer cell resistance to 5-FU [69]. In nasopharyngeal carcinoma, NNMT is associated with the phosphorylation of Akt and worse patient prognosis [70].

A recent study indicated that the overexpression of NNMT suppressed the m6A methylation of *CD44* mRNA, thereby enhancing CD44v3 formation; this process contributes to vascular invasion and distant metastasis in hepatocellular carcinoma [71]. By contrast, NNMT knockdown increased the m6A methylation of the RRACH motif (R demotes G or A; H is A, C, or U) on exon 12 and exon 19 of *CD44* mRNA [71]. The researchers suggested that NNMT-modulated CD44 m6A demethylation improves RNA stability [71]. Taken together, as NNMT, the enzyme that converts nicotinamide to MNAM, is overexpressed in a variety of human cancers, NNMT and metabolite productions are suggested to play a role in the malignant phenotype of cancer, including the involvement of the cancer stem cell phenotype.

The activity of NNMT is tightly linked to the maintenance of the NAD+ level in cells [59]. A recent study on TME via a combination of metabolomics and single-cell RNA sequencing analysis indicated that cells within ascites and ovarian cancer showed a notable enrichment in MNAM in tumor-infiltrating T cells. Of note, although MNAM levels were elevated in T cells, NNMT expression was restricted to fibroblasts and tumor cells. The study also indicated that MNAM induces T cells to secrete the tumor-promoting cytokine tumor necrosis factor-alpha. Furthermore, the study found that TME-derived MNAM can modulate T cell function and suggested that this could be a potential immunotherapy target against ovarian cancer (Figure 4B) [72].

## 7. Targeting Metabolism in TME

**Figure 5:**
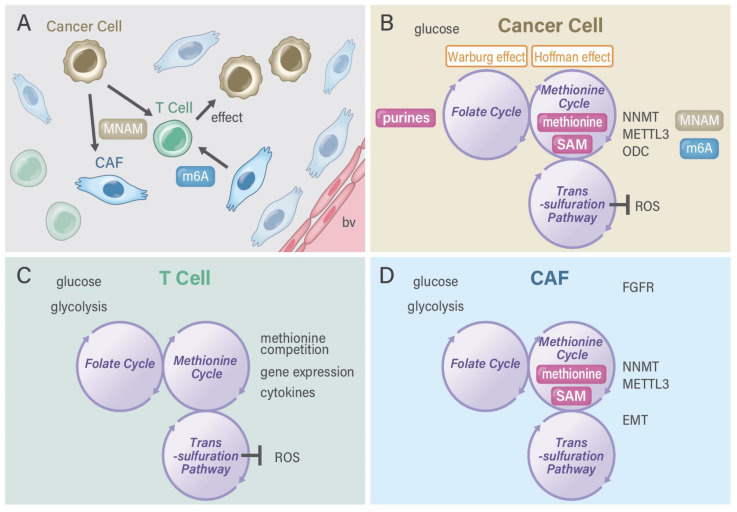
Methylosystem in the tumor microenvironment. (**A**) Various cells exist in the tumor microenvironment, including cancer cells, immune cells, and cancer-associated fibroblasts. (**B**) Cancer cells possess activated one-carbon metabolism, which comprises the folate cycle, methionine cycle, and trans-sulfuration pathway. One-carbon metabolism collaborates with the Warburg and Hoffman effects. Cancer cells can secrete microRNAs with m6A from the RNA methylation pathway and MNAM from the NAD+ salvage pathway. METTL3 function is connected with NNMT in CD44v3 cancer cells [71]. (**C**) T cells have unique redox regulation as the trans-sulfuration pathway of one-carbon metabolism plays a critical role in the maintenance and execution of T cell function [25]. (**D**) Pathway of one-carbon metabolism plays a role in maintenance of CAFs, which communicate with surrounding cells including T cells and cancer cells. CAF, cancer-associated fibroblast; ODC, ornithine decarboxylase; FGFR, fibroblast growth factor receptor; EMT, epithelial-mesenchymal transition; bv, blood vessels; MNAM, 1-methylnicotinamide.

Although studies have examined the significance and implication of CAFs, the drug discovery of CAF-targeting molecules or reagents for cancer diagnosis and therapeutic purposes has recently emerged (Table 1 and Table 2). A recent study indicated that fibroblast activation protein (FAP), which promotes tumor growth and progression, is overexpressed in the CAFs of many human epithelial cancers, including pancreatic cancer, and is an attractive target for marking by 64Cu- and 225Ac-labeled FAP inhibitor FAPI-04, which have been used as theranostics for treating FAP-expressing pancreatic cancer, as shown in a proof-of-concept study [73]. In a study on hepatocellular carcinoma, the usefulness of 68Ga-FAPI-04 positron emission tomography/computed tomography (PET/CT) was demonstrated, and 68Ga-FAPI-04 PET/CT was found to be more sensitive than 18F-FDG PET/CT in detecting hepatocellular carcinoma lesions, as 68Ga-FAPI-04 uptake is primarily correlated with tumor size, suggesting clinical benefits [74]. Another study reported that FAP-specific PET/CT imaging in fibrotic interstitial lung diseases and lung cancer provided potential clinical value for the monitoring and therapeutic evaluation of fibrotic interstitial lung diseases and suggested that these areas be investigated in future studies [75]. NNMT stabilizes sirtuin 1 in prostate cancer cells [76], whereas NNMT increases complex I activity in SH-SY5Y human neuroblastoma cells via sirtuin 3, suggesting a central role of NNMT in regulating energy homeostasis [77]. Taken together, therapy targeting FAP in the cancer stroma is effective and will contribute to the development of new treatment strategies (Figure 5).

## 8. Conclusions

Although transcription can be examined using high-speed next-generation RNA sequencing, single-cell-level analysis remains challenging for studying cancer metabolism. There is a need to clarify and understand the communication between cells across different cancers; thus, an analysis technology that is similar to single-cell analysis is required and future technological development is needed. The analysis of individual cell types is currently progressing, and as mentioned in this review, the exchange of metabolites among cells can be used to obtain an essential understanding of TME based on the methylosystem. The one-carbon metabolism-dependent modulation of nicotinamide and RNA are examples that have emerged recently as possible applications for early diagnosis and therapeutic approaches in precision medicine. Recent studies have presented increasing evidence regarding the unique metabolism of CAF-surrounding cancer cells, and CAF-targeting technology has been developed. By manipulating CAFs, it is possible to block the mechanism activating cancer cells; as a result, research expects that the approach can target CAF and modulate their function, as an efficient therapeutic strategy. Understanding such a methylosystem is expected to be an important tool in future precision medicine, such as in the development of preventive intervention methods as well as in the development of methods for early cancer diagnosis and breakthrough treatments.

## Figures and Tables

**Table 1:** NNMT and cancer.

Cancer Type	Function	References
Melanoma	Gene silencing enhances chemosensitivity	[78]
Colorectal cancer	Vanillin downregulates NNMT	[79]
HeLa cells	Inhibitor of NNMT shows antiproliferative activity	[80]
Gastric cancer	Exosomal NNMT promotes metastasis	[81]
Ovarian cancer	Low NNMT benefits from bevacizumab treatment	[82]
Gastric carcinoma	NNMT in cancer-associated fibroblasts	[83]
Hepatoblastoma	NNMT downregulation by DNA hypermethylation	[84]
Gastric cancer	Prognostic biomarker correlated with immune	[85]
Breast cancer	NNMT inhibits autophagy through AMPK pathway	[86]
Esophageal squamous carcinoma	Metabolic reprogramming and promoting the Warburg effect	[68]
Ovarian cancer	Overexpression is associated with poor prognosis	[87]
Bladder, lung, colorectal, and osteosarcoma	Cancer stem cell enrichment is associated with NNMT expression	[88]
Colorectal cancer	High stromal NNMT expression	[89]
Endometrial cancer	NNMT associates with patient survival	[90]
Skin cancer	NNMT associates with nonmelanoma skin cancers	[91]
Renal cell carcinoma	NNMT controls metabolism during progression	[92]
Cutaneous squamous cell carcinoma	NNMT induces the proliferation and invasion	[93]
Hepatocellular carcinoma	Hepatic stellate cells induce NNMT and metastasis via regulation of CD44v3	[71]
Esophageal squamous cell carcinoma	Downregulation of NNMT inhibits migration and epithelial-mesenchymal transition	[67]
Breast cancer	NNMT enhances chemoresistance through SIRT1	[94]
Ovarian cancer	NNMT is a master metabolic regulator of cancer-associated fibroblasts	[95]
Cervical carcinoma	Clinical significance of NNMT was evaluated	[96]
Oral melanoma	Potential prognostic significance	[97]
Non-Small-Cell Lung Cancer	Targeting NNMT and miR-449a in EGFR-TKI resistance	[98]
Prostate cancer	NNMT stabilizes sirtuin 1	[76]
Gastric cancer	NNMT promotes epithelial-mesenchymal transition	[66]
Melanoma	Potential involvement in tumor	[99]
Adenoid cystic carcinoma	Deregulation of NNMT and gap junction protein Alpha-1 causes metastasis	[100]
Neuroblastoma	NNMT in involved in sirtuin 3	[77]
Gastric carcinoma	A potential biomarker for worse prognosis	[101]
Renal cell carcinoma	Stage-specific changes	[102]
Pancreatic cancer	Prognostic value of NNMT in patients	[103]
Breast cancer	Downregulation of NNMT induces apoptosis via mitochondria pathway	[104]
Oral carcinoma	Silencing of NNMT inhibits tumorigenicity	[105]
Nasopharyngeal carcinoma	NNMT is associated with Akt phosphorylation and worse prognosis	[70]
Bladder cancer	Potential for a urine-based diagnostic test	[106]
Oral squamous cell carcinoma	Basis for developing a noninvasive diagnostic test	[107]
Mesenchymal cancer stem cell	Cancer stem cell NNMT enhances cellular radiation resistance	[108]
Renal cell carcinoma	NNMT activates matrix metalloproteinase-2	[109]
Glioma	Interferon-gamma elevates NNMT	[110]
Oral squamous cell carcinoma	NNMT correlates with tumor differentiation	[111]
Lung cancer	Serum levels of NNMT in patients	[112]
Hepatocellular carcinoma	NNMT is associated with poor prognosis	[113]
Bladder cancer	Metallothionein 1E and NNMT as novel regulators of cell migration	[114]
Hepatocellular carcinoma	Stat3 upregulates NNMT	[115]
Oral squamous cell carcinoma	NNMT inversely correlates with lymph node metastasis	[116]
Renal carcinoma	NNMT as a tumor marker	[117]
Colorectal cancer	Serum tumor marker	[118]
Papillary thyroid cancer	Activation of NNMT gene promoter by hepatocyte nuclear factor-1beta	[119]
Bladder cancer	Heat shock proteins and NNMT in predicting response to radiation	[120]
Colon cancer	NNMT as a marker of cancer cachexia in mice	[121]
Ehrlich ascites tumor	Preferential increase of activity of NNMT	[122]
Ehrlich ascites tumor	NNMT for malignant tumor burden	[123]

NNMT, nicotinamide N-methyltransferase; AMPK, AMP-activated protein kinase; SIRT1, Sirtuin 1 gene; EGFR-TKI, epidermal growth factor receptor-tyrosine kinase inhibitor; Stat3, signal transducer and activator of transcription 3.

**Table 2:** Recently emerged CAF-targeting FAP medicine.

Cancer Type	Drug Discovery and Application	References
Glioblastoma	Mesenchymal cells promote angiogenesis	[124]
Lung cancer	Specific PET/CT imaging	[75]
Advanced cancers	177Lu-FAPI-46	[125]
Prostate cancer	FAPI	[126]
Pancreatic cancer	68Ga-FAPI-04 PET/MR	[127]
Breast cancer	(68)Ga-FAPI-04	[127]
Diverse adenocarcinomas	(177)Lu-FAP-2286	[128]
Various cancers	Al(18)F-NOTA-FAPI	[129]
Gynecological malignancies	68Ga-FAPI-PET/CT	[130]
Lymphoma	(68)Ga-FAPI-PET/CT	[131]
Colorectal cancer	FAP binds to enolase1 and activates NF-kappaB pathway to promote metastasis	[132]
Adenoid cystic carcinomas	68Ga-FAPI-PET/CT	[133]
Sarcoma	Ga-68-FAPI	[134]
Murine HPV-positive head and neck tumors	FAP-targeted CD40 agonist (FAP-CD40)	[134]
Murine tumor models	FAP-targeted CD40 agonist induces effective antitumor immunity	[135]
Hepatocellular carcinoma	Use of nanoparticle formulation	[136]
Cancer xenografts	(4-Quinolinoyl)-glycyl-2-cyanopyrrolidine-based small molecules	[137]
Cancers	H-ferritin nanocages loaded with navitoclax	[138]
Esophageal cancer	FAP-targeted near-infrared photoimmunotherapy (NIR PIT)	[139]
Pancreatic cancer	68Ga-FAPI-PET/CT imaging	[140]
Hepatic nodules	(68)Ga-FAPI-04 PET/CT	[141]
Cancers	Liposomes bearing HER2 and FAP single-chain antibody fragments	[142]
Esophageal cancer	FAPI-PET/CT	[143]
Cancer	Bifunctional DOTA and DATA(5m) chelators	[144]
Non-small-cell lung cancer and epithelial ovarian cancer	FAP-targeted 4-1BB agonist (FAP-4-1BBL)	[145]
Head and neck cancers	FAP inhibitor PET	[146]
Cancer, heart diseases, and pulmonary fibrosis	(18)F-Labeled FAPI	[147]
Cancers	99mTc-Labeled FAPI tracers	[148]
Cancers	(68)Ga-FAPI-46 PET imaging	[149]
Pancreatic cancer xenograft mouse models	64 Cu- and 225 Ac-labeled FAPI-04	[73]
Cancers	(68)Ga-FAPI-PET/CT	[150]
Cancers	(68)Ga-FAPI-PET/CT	[151]
Cancers	Tetravalent FAP-(death receptor) DR5 antibody	[152]
Cancers	FAPI with a (4-Quinolinoyl)-glycyl-2-cyanopyrrolidine scaffold	[153]
Metastatic colorectal cancer (phase II trial)	Val-boroPro (talabostat) inhibiting FAP	[154]
Non-small-cell lung cancer	Sibrotuzumab directed against human FAP	[155]

PET, positron emission tomography; CT, computed tomography; FAP, fibroblast activation protein; FAPI, FAP inhibitor; HER2, human epidermal growth factor type 2.

## Data Availability

Not applicable.

## Abbreviations

AMPK	AMP-activated protein kinase
CAF	Cancer-associated fibroblast
CT	computed tomography
DNMT	DNA methyltransferase
EMT	epithelial-mesenchymal transition
FAP	fibroblast activation protein
FAPI	FAP inhibitor
FGFR	fibroblast growth factor receptor
EGFR-TKI	epidermal growth factor receptor
TKI	tyrosine kinase inhibitor
HER2	human epidermal growth factor type 2
HG	D-2-hydroxyglurate
HSPC	hematopoietic stem and progenitor cell
IDH	isocitrate dehydrogenase
METTL	adenosine-methyltransferase
MNAM	1-methylnicotinamide
MTHFD	methylenetetrahydrofolate dehydrogenase
NAD	Nicotinamide adenine dinucleotide
NAM	nicotinamide
NNMT	nicotinamide N-methyltransferase
ODC	ornithine decarboxylase
2PY	N-methyl-2-pyridone-5-carboxamide
4PY	N-methyl-4-pyridone-3-carboxamide
ROS	reactive oxygen species
SAH	S-adenosylhomocysteine
SAM	S-adenosylmethionine
SHMT	serine hydroxymethyltransferase
Stat	signal transducer and activator of transcription
TDH	L-threonine dehydrogenase
TME	tumor microenvironment
UPS	ubiquitin-proteasome system
PET	Positron emission tomography
PTRT	Peptide-targeted radionuclide therapy
WTAP	WT1-associated protein
YTHDF1	YT521-B homology (YTH) N6-methyladenosine RNA binding protein 1
